# Wissen zu und Umsetzung von institutionellen Schutzkonzepten in deutschen Krankenhäusern als Teil des medizinischen Kinderschutzes

**DOI:** 10.1007/s00103-024-03948-9

**Published:** 2024-09-15

**Authors:** Anna Eberhardt, Verena Sterz-Burdack, Elisa König, Jörg M. Fegert, Ulrike Hoffmann

**Affiliations:** Klinik für Kinder- und Jugendpsychiatrie/Psychotherapie, Uniklinikum Ulm, Steinhövelstr. 5, 89075 Ulm, Deutschland

**Keywords:** Gewaltexposition, Kindeswohl, Prävention und Eindämmung von Krankheiten, Kinder und Jugendliche, kurzfristig stationär untergebracht, Kinder und Jugendliche, langfristig stationär untergebracht, Exposure to violence, Child welfare, Prevention and control, Children and adolescents, hospitalized, Children and adolescents, institutonalized

## Abstract

**Hintergrund:**

Aktuelle Studien zeigen die hohe Prävalenz von Kindesmisshandlung in Deutschland und dass Übergriffe durch Krankenhauspersonal ebenfalls ein Problem darstellen. Seit 2020 fordert der Gemeinsame Bundesausschuss institutionelle Schutzkonzepte (ISK) zum Schutz und zur Unterstützung von Betroffenen in Kliniken. Diese Untersuchung hat zum Ziel, zu analysieren, wie hoch Wissen und Handlungskompetenzen zu Kindesmisshandlungen bei Krankenhausmitarbeitenden sind und inwiefern ISK in Krankenhäusern umgesetzt werden.

**Methoden:**

Es wurden 1011 Teilnehmende vor dem Belegen zweier Online-Kurse zu Kinderschutz und Schutzmaßnahmen in Krankenhäusern zu selbsteingeschätztem Wissens- und Kompetenzstand im Kinderschutz und Auftreten von Misshandlungsfällen und Schutzmaßnahmen in der eigenen Einrichtung befragt. Die Daten wurden mittels deskriptiver Verfahren ausgewertet.

**Ergebnisse:**

Es zeigt sich, dass Wissen und Kompetenzen von Krankenhausmitarbeitenden zu Kinderschutz im Mittelfeld liegen. Besonders häufig werden in Krankenhäusern Misshandlungsfälle wahrgenommen, die außerhalb der Institution begangen werden, aber auch Gewalt durch Mitarbeitende bzw. gleichaltrige Mitpatient*innen spielt eine bedeutende Rolle. 93,6 % der Befragten gaben an, dass ihre Einrichtung bereits mindestens ein Element eines ISK entwickelt habe, allerdings berichteten nur 1,0 % der Befragten, dass bereits alle abgefragten Elemente vollständig entwickelt wurden.

**Diskussion:**

Krankenhäuser in Deutschland scheinen sich überwiegend auf den Weg gemacht zu haben, Kinder und Jugendliche besser vor Misshandlung zu schützen und Betroffene zu unterstützen. Allerdings bestehen nach wie vor noch Defizite bei den Kompetenzen der Mitarbeitenden und der Umsetzung der ISK-Elemente. Hierfür müssen mehr Ressourcen von den Trägern der Krankenhäuser und von der öffentlichen Hand zur Verfügung gestellt werden.

## Abstract (en)

**Background:**

Recent studies show the high prevalence of child maltreatment in Germany and that assaults by hospital staff also pose a problem. Since 2020, the Joint Federal Committee has been calling for institutional safeguarding measures (ISM) to protect and support those affected in hospitals. The aim of this study is to analyze the level of knowledge and action competencies regarding child maltreatment among hospital staff and the extent to which ISM are implemented in hospitals.

**Methods:**

A survey was administered to 1011 participants before they took part in two online courses on child protection and protection measures in hospitals, with questions regarding self-assessed level of knowledge and competence on child protection and the occurrence of cases of maltreatment and protective measures in their own institution. Data were analyzed using descriptive methods.

**Results:**

It becomes apparent that the knowledge and competencies of hospital staff regarding child protection are in the middle range. In hospitals, cases of maltreatment that occur outside the institution are particularly common, but violence by staff or other patients of the same age also plays a significant role. Of the respondents, 93.6% stated that their institution had already developed at least one element of an ISM, but only 1.0% of respondents reported that all the elements surveyed had already been fully developed.

**Discussion:**

Hospitals in Germany seem to be largely on the path to better protect children and adolescents from maltreatment and to support those affected. However, there are still deficits in the competencies of employees and the implementation of the ISM elements. More resources need to be made available by hospital operators and the public sector for this purpose.

## Hintergrund

Misshandlungserfahrungen in Kindheit und/oder Jugend haben ein hohes Risiko für somatische, psychische und soziale Folgeerscheinungen auch im Erwachsenenalter [1–5]. Daneben bringen die Folgen von Kindesmisshandlung auch enorme volkswirtschaftliche Kosten mit sich [6]. Kindesmisshandlung wird definiert als jede Handlung oder Unterlassung, die zu einer (potenziellen) Schädigung führt oder diese androht [7]. Basierend auf dieser Definition werden unter Kindesmisshandlung die Formen emotionale und körperliche Misshandlung, sexueller Missbrauch/sexualisierte Gewalt und Vernachlässigung subsumiert. Kindesmisshandlung findet oftmals im nahen sozialen Umfeld statt, kann aber auch an sonstigen Aufenthalts- und Lebensorten von Kindern und Jugendlichen, wie etwa im institutionellen Kontext, passieren. Die Anzahl der im Hellfeld wahrgenommenen Kindeswohlgefährdungen hat 2022 mit fast 62.300 betroffenen Kindern und Jugendlichen einen neuen Höchststand erreicht [8]. Aufgrund ihrer umfassenden Folgen und ihrer Häufigkeit sollte Kindesmisshandlung als ein relevantes Problem der öffentlichen Gesundheit anerkannt werden. Präventive Maßnahmen wie Schulungen, Sensibilisierung und die Entwicklung eines institutionellen Schutzkonzeptes (ISK) sind wichtig, um Kinder und Jugendliche zu schützen und die Folgen von Misshandlung abzuschwächen.

Medizinische Einrichtungen wie Kliniken und Krankenhäuser, in welchen Kinder und Jugendliche betreut und behandelt werden, stellen für die Zeit des Aufenthalts wichtige Räume des Aufwachsens und Genesens für Kinder und Jugendliche dar. In der Altersgruppe der unter 15-Jährigen kam es 2021 beispielsweise zu 13.809 stationären Behandlungen in Krankenhäusern je 100.000 Einwohner*innen [9]. Im Rahmen der Behandlung von Kindern und Jugendlichen in Krankenhäusern kommt es auch zu Gewalt durch Fachkräfte [10, 11]. So zeigte beispielsweise eine für Deutschland bevölkerungsrepräsentative Studie, dass 19 % der befragten Kindern und Jugendlichen bei stationären Aufenthalten in medizinischen Einrichtungen mindestens eine Art von Misshandlung durch das Pflegepersonal erlebt haben [12]. Zudem bestehen in Kliniken und Krankenhäusern besondere Risiken im Hinblick auf Kindesmisshandlung, die sich beispielsweise aus der Notwendigkeit körperlicher Untersuchungen, dem teils längeren Aufenthalt von Kindern oder Jugendlichen ohne Bezugspersonen in einer für sie fremden Umgebung und den Vorerfahrungen einiger Kinder und Jugendlicher mit Gewalt ergeben [13].

Die Fachkräfte in medizinischen Einrichtungen stellen auf der anderen Seite für Kinder und Jugendliche, welche von Gewalt zum Beispiel innerhalb der Familie betroffen sind, oftmals wichtige Ansprechpersonen dar. Betrachtet man die deutschlandweite Prävalenz von Kindesmisshandlung von 31 % [14], zeigt sich, wie hoch die Wahrscheinlichkeit ist, dass Fachkräfte in medizinischen Einrichtungen in ihrem Arbeitsalltag mit Fällen von Kindesmisshandlung zu tun haben. Allerdings fehlt es medizinischen Fachkräften im Umgang mit einem Verdachtsfall oft an Wissen und Kompetenzen, was zu einem Übersehen, Nichternstnehmen oder einer inadäquaten Behandlung von diesen Fällen führen kann. Somit kann es auch zu einer nachteiligen Auwirkung auf den institutionellen Kinderschutz, also die Maßnahmen und Strategien, die von Institutionen, die mit Kindern arbeiten, ergriffen werden können, um Kinder vor Missbrauch, Vernachlässigung und anderen Formen von Gewalt und Gefährdung zu schützen, kommen [15–17].

Seit 2020 sieht der Gemeinsame Bundesausschuss (G-BA) Regelungen zu ISK in Kliniken, Krankenhäusern und ambulanten Einrichtungen in seiner Qualitätsmanagement-Richtlinie vor [18]. Ein ISK besteht dabei aus mehreren Elementen, welche sich den Ebenen Analyse, Prävention, Intervention und Aufarbeitung zuordnen lassen. Diese Elemente bilden einen Rahmen, der jedoch von jeder Institution spezifisch inhaltlich gefüllt und angepasst werden muss [19]. Kliniken und Krankenhäuser stehen somit in der Pflicht, dafür zu sorgen, dass sie einen Schutz- und Kompetenzort für die ihnen anvertrauten Kinder und Jugendlichen darstellen. Das bedeutet zum einen, dass institutionelle Strukturen und Abläufe so gestaltet werden müssen, dass Grenzüberschreitungen erkannt, benannt und Maßnahmen ergriffen werden, diese zu stoppen bzw. zu verhindern (Schutzort), sowie zum anderen, Kindern und Jugendlichen, die von (sexualisierter) Gewalt betroffen sind, in der Institution Unterstützung und Hilfe zu bieten (Kompetenzort). Eine Untersuchung zum Stand der Umsetzung von ISK im Gesundheitsbereich zeigte 2019, dass von den 165 untersuchten Kliniken nur 15,8 % ein komplettes ISK mit allen Elementen entwickelt hatten [13].

Die Deutsche Krankenhausgesellschaft (DKG) ermöglichte es daher allen Mitarbeitenden ihrer Mitgliedsverbände, von 2019 bis 2022 kostenlos an 2 Online-Kursen zur Entwicklung eines ISK („Schutzkonzepte in Organisationen – Schutzprozesse partizipativ und achtsam gestalten“ und „Leitungswissen Kinderschutz in Institutionen – ein Online-Kurs für Führungskräfte“) teilzunehmen, welche von 2014–2017 unter der Förderung des Bundesministeriums für Bildung und Forschung (BMBF) an der Klinik für Kinder- und Jugendpsychiatrie/Psychotherapie des Universitätsklinikums Ulm entwickelt wurden.

Ziel dieser Untersuchung ist es, Wissen und Kompetenzen bei Fachkräften im institutionellen Kinderschutz und die Häufigkeit von Verdachtsfällen auf Kindesmisshandlung, die außerhalb der medizinischen Einrichtung, also zum Beispiel im privaten Umfeld (externe Fälle), und innerhalb der Einrichtungen durch Mitarbeitende oder Mitpatient*innen (interne Fälle) stattfinden, zu erfassen sowie die Umsetzung und Akzeptanz von ISK-Elementen in Kliniken in Deutschland zu analysieren.

## Methoden

### Teilnehmende und Vorgehen

Von 2019 bis 2022 konnten Mitarbeitende an Kliniken, die Mitglied in einem Mitgliedsverband der Deutschen Krankenhausgesellschaft waren, kostenfrei an 2 Online-Kursen zum Thema Kinderschutz in Institutionen teilnehmen. Der Online-Kurs „Schutzkonzepte in Organisationen – Schutzprozesse partizipativ und achtsam gestalten“ hatte dabei zum Ziel, Ansätze und Anstöße für die Entwicklung und Etablierung eines ISK in der eigenen Organisation zu vermitteln, und richtete sich an Mitarbeitende von Organisationen, die Verantwortung für Kinder und Jugendliche tragen. Der Online-Kurs „Leitungswissen Kinderschutz in Institutionen – ein Online-Kurs für Führungskräfte“ richtete sich an Leitungskräfte von Einrichtungen, in denen Kinder und/oder Jugendliche betreut werden, und sollte die Kursteilnehmenden darin unterstützen, den Prozess der ISK-Entwicklung in der Institution anzuleiten und entsprechende Kenntnisse zu erwerben. Die Studienstichprobe mit 1011 Proband*innen setzt sich aus 591 Teilnehmenden des Online-Kurses „Schutzkonzepte in Organisationen – Schutzprozesse partizipativ und achtsam gestalten“ und 420 Teilnehmenden des Online-Kurses „Leitungswissen Kinderschutz in Institutionen – ein Online-Kurs für Führungskräfte“ zusammen.

### Datenerhebung

Die Teilnehmenden der beiden Online-Kurse mussten vor Beginn der Kurse einen Online-Fragebogen zu selbsteingeschätztem Wissens- und Kompetenzstand zu institutionellem Kinderschutz und der Umsetzung eines ISK im eigenen Krankenhaus ausfüllen, um Zugang zu den Kursinhalten zu bekommen. Begleitend wurden ihre demografischen und berufsspezifischen Daten erhoben. Sonstige Berufsgruppen und Arbeitsplätze bzw. nichtmedizinische Arbeitsplätze wurden bei berufsgruppen- und arbeitsplatzspezifischen Auswertungen nicht miteinbezogen. Konstrukte zu Wissen und Handlungskompetenz im institutionellen Kinderschutz sowie zur Umsetzung und Akzeptanz von ISK-Elementen wurden in beiden Kursen mit den gleichen Items erhoben. Die Teilnehmenden am Online-Kurs „Leitungswissen Kinderschutz in Institutionen – ein Online-Kurs für Führungskräfte“ wurden zudem gefragt, ob sie in ihrer Rolle als Leitungskraft innerhalb der letzten 3 Jahre mit (Verdachts‑)Fällen von sexueller, körperlicher oder psychischer Kindesmisshandlung konfrontiert waren, und wenn ja, gegen wen der entsprechende Verdacht bestand (externe Personen oder Patient*innen oder Mitarbeitende innerhalb des Krankenhauses).

Die Items zu Wissen und Handlungskompetenz zu institutionellem Kinderschutz, Umsetzungsstand und der Akzeptanz der ISK-Elemente wurden über Selbsteinschätzungen auf 6‑ bzw. 4‑stufigen Endpunktskalen erhoben (Tab. 1).

**Tab. 1:** Übersicht der Erhebungsitems und Kategorien der dazugehörigen Endpunktskalen

Item	Skala					
	Endpunkt					Endpunkt
	1 (sehr gering)	2	3	4	5	6 (sehr umfangreich)
Wie schätzen Sie Ihr Wissen zum Thema „institutioneller Kinderschutz“ ein?	○	○	○	○	○	○
Wie schätzen Sie Ihre Handlungskompetenzen bezüglich des Themas „Institutioneller Kinderschutz“ ein?	○	○	○	○	○	○
	1 (hoch)	2		3		4 (niedrig)
Wie hoch schätzen Sie die Akzeptanz des [Schutzkonzeptelements] bei Ihren Mitarbeiter*innen bzw. Kolleg*innen ein?	○	○		○		○
Wie schätzen Sie den Umsetzungsgrad des [Schutzkonzeptelements] bei Ihren Mitarbeiter*innen bzw. Kolleg*innen ein?	○	○		○		○

Die abgefragten Elemente eines ISK orientierten sich an den von Hoffmann und Kolleg*innen definierten Elementen eines Schutzkonzeptes im medizinischen Bereich, welche auf den Empfehlungen des Runden Tisches Sexueller Kindesmissbrauch und den Vorschlägen der Unabhängigen Beauftragten für Fragen des sexuellen Kindesmissbrauchs beruhen [19–21]. Abgefragt wurde somit die Umsetzung von Risikoanalyse, Verhaltenskodex, Leitbild, Interventionsplan, Rehabilitationsverfahren und Handlungsempfehlungen zur Aufarbeitung von (Verdachts‑)Fällen. Im Bereich der kinderschutzsensiblen Gewinnung von Mitarbeitenden wurde nach der Einholung des erweiterten polizeilichen Führungszeugnisses (EFZ) und von Selbstverpflichtungserklärungen gefragt. Zudem wurden allgemeine Präventionsmaßnahmen und die Aufklärung über Kinderrechte im Speziellen erhoben. Pädagogische Konzepte im Allgemeinen und speziell für den Bereich Sexualpädagogik und Beschwerdeverfahren/Partizipationsmöglichkeiten für Kinder/Jugendliche, Mitarbeitende und externe Erwachsene (z. B. Eltern) wurden zusätzlich abgefragt. Die Befragten konnten jeweils zwischen den Antworten „vorhanden bzw. wird im Moment durchgeführt“, „nicht vorhanden“, „nicht vorhanden, aber in Planung“, „nicht sicher“ und „für meine Einrichtung nicht relevant“ (z. B. pädagogisches Konzept in einer Klinik für Erwachsenenpsychiatrie) wählen. Für die vorliegende Arbeit wurde nur ausgewertet, wenn die Befragten angekreuzt haben, dass ein Element vorhanden ist bzw. sich in Durchführung befindet.

### Datenanalyse

Das Wissen und die Handlungskompetenz im institutionellen Kinderschutz wurden deskriptiv mittels Boxplots beschrieben, um die Verteilung der Streuungs- und Lagemaße grafisch aufzuzeigen. Auch die Darstellung der berichteten (Verdachts‑)Fälle in den Krankenhäusern und die Realisation der ISK-Elemente wurden deskriptiv mittels bivariater Häufigkeitsanalysen untersucht, um die Häufigkeitsverteilungen der verschiedenen Merkmale über die verschiedenen Einrichtungen hinweg betrachten zu können. Hierfür erfolgt die Angabe der absoluten und relativen Häufigkeiten. Bei Umsetzungsstand und Akzeptanz des ISK wurde mittels Mittelwert und Standardabweichung die zentrale Tendenz der Verteilung dargestellt. Die Berechnungen wurden mit der Statistik- und Analysesoftware SPSS (Version 29.0, IBM, Armonk, NY, USA) durchgeführt [22].

## Ergebnisse

Insgesamt nahmen 1011 Fachkräfte an den Online-Fortbildungen teil und füllten die Befragung vor Start der Kurse aus. Das Durchschnittsalter der Teilnehmenden lag bei 43 Jahren, die durchschnittliche Berufserfahrung bei 13 Jahren. Dreiviertel der Teilnehmenden waren Frauen, lediglich ein Viertel Männer. Die größte Berufsgruppe waren Ärzt*innen gefolgt von pädagogischem Personal (z. B. Pädagog*innen, Sozialpädagog*innen/Sozialarbeiter*innen, Heilpädagog*innen, Erzieher*innen). Fast die Hälfte der Befragten arbeitete in einer Klinik für Kinder- und Jugendpsychiatrie, -psychotherapie oder -psychosomatik, circa ein Viertel in einer Klinik für Kinder- und Jugendmedizin (Tab. 2). Den Online-Kurs „Schutzkonzepte in Organisationen – Schutzprozesse partizipativ und achtsam gestalten“ belegten 591 (58,5 %) der Teilnehmenden und 420 (41,5 %) den Online-Kurs „Leitungswissen Kinderschutz in Institutionen – ein Online-Kurs für Führungskräfte“.

**Tab. 2:** Beschreibung der Stichprobe (*N* = 1011)

		MW	SD
**Alter [Jahre] **(*n* = 1009)		43,42	10,81
**Berufserfahrung [Jahre]**		12,56	9,72
		***n***	**%**
**Geschlecht**			
*Weiblich*		760	75,2
*Männlich*		251	24,8
**Berufsgruppe**			
Ärzt*in		310	30,7
Psychotherapeut*in		126	12,5
Psychotherapeut*in in Ausbildung		67	6,6
Psycholog*in		35	3,5
*Pädagogisches Personal*		213	21,1
*Pflegepersonal*		177	17,5
Co-Therapeut*in		19	1,9
*Verwaltung*		56	5,5
*Sonstige*		8	0,8
**Arbeitsplatz: Klinik für**			
*Kinder- und Jugendpsychiatrie/-psychotherapie/-psychosomatik*		487	48,5
*Psychiatrie/Psychotherapie/Psychosomatik (Erwachsene)*		42	4,2
*Kinder- und Jugendmedizin*		257	25,4
*Andere Klinik*		149	14,7
*Sonstige*		16	1,6
*Nichtmedizinischer Arbeitsplatz*		60	5,9

*MW* Mittelwert; *SD* Standardabweichung

### Häufigkeit von Kindesmisshandlungen in Institutionen

**Abb. 1:**
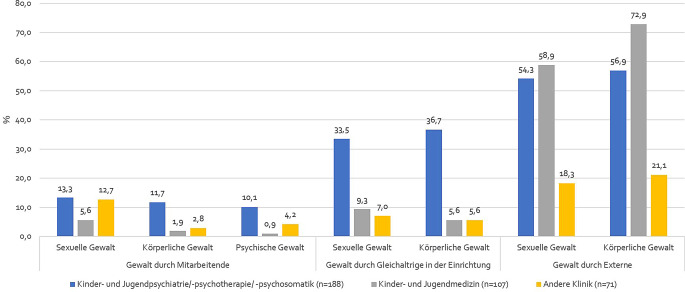
Wahrnehmung von (Verdachts‑)Fällen sexueller, körperlicher oder psychischer Kindesmisshandlung von Befragten aus dem Online-Kurs „Leitungswissen Kinderschutz in Institutionen – ein Online-Kurs für Führungskräfte“ durch Mitarbeitende oder Gleichaltrige innerhalb der Einrichtung und durch Personen außerhalb der Einrichtung getrennt nach Art des Krankenhauses in % bezogen auf die jeweilige Einrichtung (*n* = 381). *Quelle*: eigene Abbildung

Die Teilnehmenden des Online-Kurses „Leitungswissen Kinderschutz in Institutionen – ein Online-Kurs für Führungskräfte“ wurden nach dem Auftreten von (Verdachts‑)Fällen in der eigenen Institution befragt. Abb. 1 zeigt, dass (Verdachts‑)Fälle von Kindesmisshandlung, die außerhalb des Krankenhauses aufgetreten sind, besonders häufig geschildert wurden. Mehr als die Hälfte der Befragten aus dem Bereich der Kinder- und Jugendpsychiatrie/-psychotherapie/-psychosomatik und Kinder- und Jugendmedizin berichtete von (Verdachts‑)Fällen sexueller Kindesmisshandlung außerhalb des Krankenhauses, noch mehr solcher externen (Verdachts‑)Fälle wurden bei körperlicher Kindesmisshandlung berichtet. Blickt man auf die Häufigkeit von Gewalt innerhalb von Krankenhäusern (interne Fälle), fällt auf, dass vor allem in Einrichtungen der Kinder- und Jugendpsychiatrie/-psychotherapie/-psychosomatik ein Drittel der Befragten von (Verdachts‑)Fällen sexueller und körperlicher Gewalt durch Gleichaltrige berichtete. In den anderen Kliniken bleiben die Werte im einstelligen Prozentbereich. (Verdachts‑)Fälle sexuelle Kindesmisshandlung durch Mitarbeitende gaben 13,3 % der Mitarbeitenden aus der Kinder- und Jugendpsychiatrie/-psychotherapie/-psychosomatik und 12,7 % aus anderen Kliniken an. Das Auftreten von (Verdachts‑)Fällen körperlicher Gewalt durch Mitarbeitende des Krankenhauses gaben 11,7 %, das Auftreten von (Verdachts‑)Fällen psychischer Kindesmisshandlung durch Mitarbeitende gaben 10,1 % der Befragten aus der Kinder- und Jugendpsychiatrie/-psychotherapie/-psychosomatik an.

### Wissen und Handlungskompetenz bei medizinischem Fachpersonal zu institutionellem Kinderschutz

**Abb. 2:**
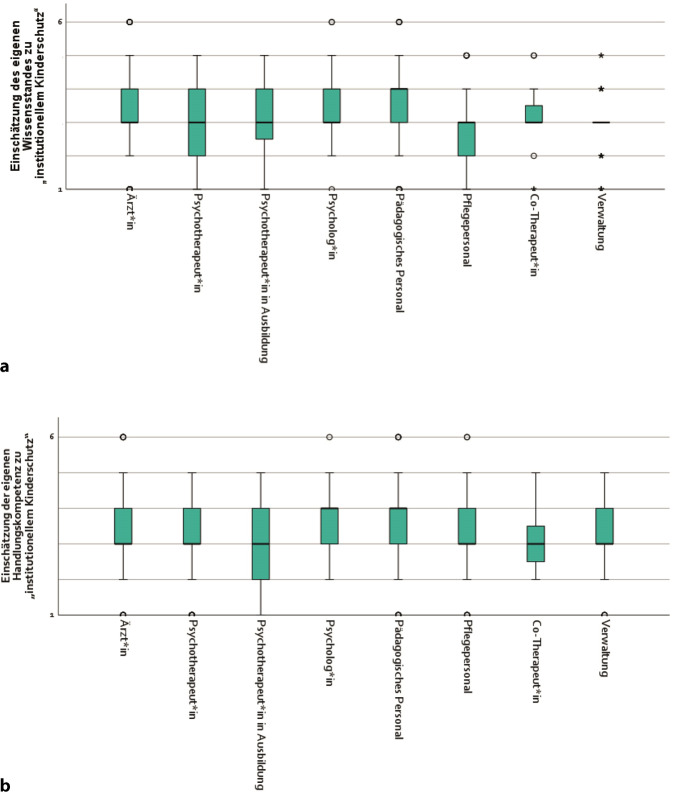
Selbsteinschätzung zu Wissensstand (**a**) und Handlungskompetenz (**b**) bzgl. institutionellem Kinderschutz unter medizinischem Fachpersonal, getrennt nach Berufsgruppen, zentrale Tendenz und Streuung (gemessen mit 6‑stufiger Skala: 1 = sehr gering bis 6 = sehr umfangreich) (*n* = 1003). *Quelle*: eigene Abbildung

Bei der Befragung der Teilnehmenden zur Selbsteinschätzung ihres Wissens im Bereich des institutionellen Kinderschutzes befand sich der Median über fast alle Berufsgruppen hinweg im mittleren Bereich der 6‑stufigen Skala (Abb. 2a). Pädagogisches Personal war mit einem Median im oberen Bereich und dem 25 %-Quartil im mittleren Bereich der Skala die Ausnahme. Bei Psychotherapeut*innen approbiert und in Ausbildung und Pflegekräften lagen die 25 %-Quartile am unteren Ende der Skala.

Im Vergleich zum Wissensstand bei institutionellem Kinderschutz zeigte sich bei der Handlungskompetenz im institutionellen Kinderschutz ein etwas positiveres Bild. Der Median lag bei den Ärzt*innen, Psychotherapeut*innen, sowohl approbierte als auch in Ausbildung befindliche, Pflegefachkräften und Verwaltungsmitarbeitenden weiterhin im mittleren Bereich der 6‑stufigen Skala (Abb. 2b). Psycholog*innen und pädagogisches Personal zeigten allerdings einen höheren Median. Bei Psychotherapeut*innen in Ausbildung und Co-Therapeut*innen lagen die 25 %-Quartile jedoch im unteren Bereich der Skala. Bei allen anderen Berufsgruppen befand sich der Interquartilsabstand (IQR) aber in der oberen Hälfte der Skala.

### Prävention und Schutzmaßnahmen

**Abb. 3:**
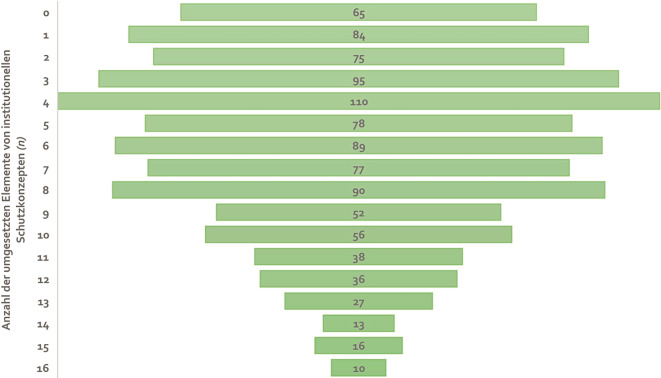
Anzahl von Elementen eines institutionellen Schutzkonzeptes (ISK), die in den Einrichtungen der Befragten bereits umgesetzt wurden (*n* = 1011). *Quelle*: eigene Abbildung

Im Hinblick auf die Entwicklung von institutionellen Präventions- und Schutzmaßnahmen gaben 93,6 % der Befragten (*n* = 946) an, dass ihre Einrichtung bereits mindestens ein Element eines ISK entwickelt habe, 65 Befragte (6,4 %) gaben dagegen für kein einziges der genannten Elemente eines ISK an, dass es in ihrer Einrichtung bereits entwickelt wurde (Abb. 3). Am häufigsten wurde angegeben, dass bereits 4 Elemente entwickelt wurden (*n* = 110; 10,9 %). Lediglich 10 Befragte (1,0 %) gaben an, dass in ihrer Einrichtung bereits alle 16 Elemente realisiert wurden.

**Abb. 4:**
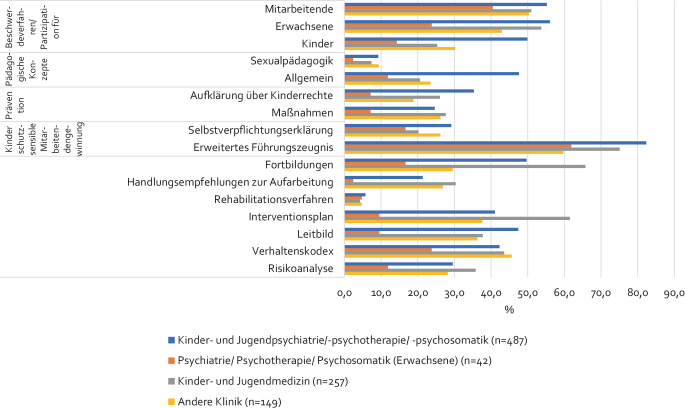
Realisation der abgefragten 16 Elemente institutioneller Schutzkonzepte (ISK) getrennt nach Einrichtung der Befragten (*n* = 935). *Quelle*: eigene Abbildung

Blickt man getrennt nach Einrichtungen auf die verschiedenen Elemente eines ISK zeigt sich, dass Mitarbeitende von Kliniken für Kinder- und Jugendpsychiatrie/-psychotherapie/-psychosomatik und der Kinder- und Jugendmedizin am häufigsten angaben, dass bereits Elemente eines ISK in ihrer Einrichtung realisiert wurden. Die Mitarbeitenden von Kliniken der Psychiatrie/Psychotherapie/Psychosomatik für Erwachsene gaben am seltensten an, dass Elemente realisiert wurden (Abb. 4). Die Mehrheit der Teilnehmenden gab an, dass ein EFZ von den Mitarbeitenden eingeholt wurde, viele berichteten zudem über Beschwerde- bzw. Partizipationsmöglichkeiten für Kinder/Jugendliche bzw. Erwachsene, einen Interventionsplan im Verdachtsfall, ein Klinikleitbild, einen Verhaltenskodex und Fortbildungen zur Thematik. Sexualpädagogische Konzepte und Verfahren zur Rehabilitation von beschuldigten Mitarbeitenden wurden in den Einrichtungen aller Befragten bisher nur selten realisiert.

Für die Elemente Verhaltenskodex, Leitbild, pädagogisches Konzept, sexualpädagogisches Konzept und Interventionsplan wurde dann zusätzlich betrachtet, inwieweit diese Elemente bereits umgesetzt wurden und wie hoch die Befragten die Akzeptanz der Elemente bei den anderen Mitarbeitenden einschätzen. Tab. 3 zeigt, dass die Angaben zu Umsetzung und Akzeptanz der verschiedenen Elemente im mittleren bis hohen Bereich lagen. Mitarbeitende von Kliniken der Psychiatrie/Psychotherapie/Psychosomatik für Erwachsene schätzten Umsetzung und Akzeptanz der Elemente insgesamt etwas schlechter ein als Mitarbeitende der anderen Kliniken.

**Tab. 3:** Umsetzungsstand und Akzeptanz von Elementen institutioneller Schutzkonzepte (ISK) im Mittel, getrennt nach Einrichtung der Befragten (gemessen mit 4‑stufiger Skala: 1 = hoch bis 4 = niedrig)

Klinik	Verhaltenskodex		Leitbild		Pädagogisches Konzept		Sexualpädagogisches Konzept		Interventionsplan	
	U	A	U	A	U	A	U	A	U	A
	MW; SD (*n*)	MW; SD (*n*)	MW; SD (*n*)	MW; SD (*n*)	MW; SD (*n*)	MW; SD (*n*)	MW; SD (*n*)	MW; SD (*n*)	MW; SD (*n*)	MW; SD (*n*)
**Kinder- und Jugendpsychiatrie/-psychotherapie/-psychosomatik**	1,9; 0,6 (206)	1,7; 0,6 (206)	1,9; 0,6 (231)	1,7; 0,6 (231)	1,8; 0,6 (232)	1,8; 0,6 (232)	2,1; 0,7 (45)	1,8; 0,6 (45)	1,9; 0,7 (200)	1,7; 0,6 (200)
**Psychiatrie/Psychotherapie/Psychosomatik (Erwachsene)**	2,3; 0,7 (10)	2,3; 0,8 (10)	1,8; 0,5 (4)	2,0; 0,8 (4)	1,8; 1,3 (5)	1,8; 1,3 (5)	^#^	^#^	2,0; 0,8 (4)	2,0; 0,8 (4)
**Kinder- und Jugendmedizin**	1,9; 0,5 (112)	1,7; 0,5 (112)	1,9; 0,7 (97)	1,7; 0,6 (97)	1,8; 0,6 (53)	1,7; 0,6 (53)	1,9; 0,8 (19)	1,7; 0,7 (19)	1,9; 0,7 (158)	1,7; 0,6 (158)
**Andere Klinik**	1,9; 0,5 (68)	1,8; 0,6 (68)	1,7; 0,6 (54)	1,6; 0,6 (54)	1,7; 0,6 (35)	1,5; 0,5 (35)	1,7; 0,7 (14)	1,6; 0,7 (14)	1,9; 0,7 (56)	1,7; 0,6 (56)

*MW* Mittelwert, *SD* Standardabweichung, *U* Grad der Umsetzung, *A* Akzeptanz, ^#^ keine Berechnung möglich, da *n* = 1

## Diskussion

Ziel der Studie war es, zu untersuchen, wie häufig Fachkräfte in Kliniken und Krankenhäusern mit externen und internen Fällen von Kindesmisshandlung konfrontiert werden, wie gut ihr Wissen und ihre Handlungskompetenzen hierzu sind und inwieweit ISK-Elemente in Krankenhäusern und Kliniken bereits umgesetzt und akzeptiert werden. Insgesamt lagen Wissen und Handlungskompetenzen der Mitarbeitenden im Mittelfeld. Besonders oft traten in Krankenhäusern und Kliniken externe Fälle von Misshandlung zu Tage. Die Mehrheit der Befragten gab an, dass ihre Einrichtung bereits mehrere Elemente eines ISK entwickelt hat, allerdings berichteten nur sehr wenige der Befragten, dass die ISK-Entwicklung bereits abgeschlossen ist.

Auffällig bei den vorliegenden Ergebnissen ist, dass die Befragten vor allem von externen Fällen berichteten, die in ihrer Einrichtung behandelt wurden. Die Befragten der Kinder- und Jugendpsychiatrie/-psychotherapie/-psychosomatik berichteten in diesem Zusammenhang vermehrt auch von Fällen von Gewalt durch Kinder und Jugendliche. Die häufige Wahrnehmung von Misshandlungsfällen, die außerhalb des Krankenhauses stattfinden, kann sich durch die Behandlung der medizinischen Folgen von Misshandlungserfahrungen in Krankenhäusern erklären lassen und zeigt nochmals die bedeutende Rolle von Krankenhäusern und deren Personal bei der Unterstützung von Betroffenen (Kompetenzort), welche bereits in vergangenen Untersuchungen immer wieder erwähnt wurde [13, 23, 24]. Zudem könnte auch eine Hypothese sein, dass es „bequemer“ ist, externe statt interner Gewalt wahrzunehmen. Beim Umgang mit externen Fällen stellen sich der Fachkraft jedoch auch besondere Herausforderungen, wie Kenntnisse zur Anamnese im Kinderschutz und zum Vorgehen bei Verdachtsfällen, zum Umgang mit Angehörigen und Beschuldigten sowie zur Arbeitsweise von Kinderschutzgruppen. Die Gewalt durch Gleichaltrige in Kliniken der Kinder- und Jugendpsychiatrie/-psychotherapie/-psychosomatik kann wahrscheinlich auch auf die Behandlung von Kindern und Jugendlichen mit Krankheitsbildern, die zum Beispiel vermehrt aggressives Verhalten mit sich bringen, in diesen Einrichtungen zurückgeführt werden.

Gewalt durch Mitarbeitende wurde von den Befragten zwar am seltensten berichtet, auch hier befanden sich die Werte aber für alle Gewaltformen teilweise im 2‑stelligen Prozentbereich. Dieses Ergebnis ähnelt den Ergebnissen einer für Deutschland bevölkerungsrepräsentativen Studie zu Gewalt durch Pflegekräfte, die zeigen konnte, dass bei stationären Aufenthalten in medizinischen Einrichtungen 19 % der Befragten mindestens eine Art von Misshandlung in Kindheit und Jugend durch Pflegepersonal in Krankenhäusern erlitten haben [12]. Bei einer weiteren Untersuchung von Weidner aus dem Jahr 2017 gab fast ein Drittel der befragten Pflegekräfte an, dass Maßnahmen gegen den Willen von Patient*innen üblich sind. Ein Anteil von 11,5 % der Befragten gab an, dass Gewalt gegenüber Patient*innen häufig vorkommt [10]. In einer repräsentativen telefonischen Befragung von 250 stellvertretenden Pflegedienstleitungen in stationären Einrichtungen gaben zudem fast die Hälfte der Befragten an, dass sie Konflikte, Aggression und Gewalt in der Pflege als ein wesentliches Problem sehen [11]. Zu Übergriffen durch Pflegekräfte und anderes Personal in Krankenhäusern ist die Studienlage aktuell allerdings noch sehr dünn. Da die bisherigen Ergebnisse aber nahelegen, dass dies ein bedeutendes Problem darstellt, sollten weitere Untersuchungen durchgeführt werden.

In der vorliegenden Studie wurde des Weiteren deutlich, dass der Wissensstand bei den Mitarbeitenden von Krankenhäusern zu Kindesmisshandlungen meist lediglich im Mittelfeld liegt. Vor allem bei den Pflegefachkräften zeigten sich niedrigere Werte. Die Handlungskompetenz wurde von den Teilnehmenden etwas besser eingeschätzt als der eigene Wissensstand zu Kindesmisshandlungen. Hier zeigte sich vor allem, dass diejenigen, die sich noch in der Ausbildung befinden, größere Unsicherheiten haben. Insgesamt stimmten die Werte mit der aktuellen Studienlage zum Thema überein. So haben bereits andere Untersuchungen zeigen können, dass der Wissens- und Kompetenzstand im medizinischen Kinderschutz unter Gesundheitsfachkräften eher niedrig eingeschätzt wird und dies oftmals zu Unsicherheiten und Ängsten führt [15–17, 25, 26]. Zudem wird das Thema in den Ausbildungen der verschiedenen Fachkräfte oftmals nicht thematisiert, was Unsicherheiten von Auszubildenden erklären kann [27]. In den vergangenen Jahren konnte das Thema Kindesmisshandlung mehr Aufmerksamkeit auch im medizinischen Bereich erlangen, z. B. durch die Gründung der Arbeitsgemeinschaft und späteren Deutschen Gesellschaft für Kinderschutz in der Medizin (DGKiM) und ihre Schulungs- und Fortbildungstätigkeiten, die International Statistical Classification of Diseases and Related Health Problems (ICD) und den Operationen- und Prozedurenschlüssel (OPS) zur Kodierung von Misshandlungsfällen und deren Diagnostik, die AWMF S3(+) Kinderschutzleitlinie, das Inkrafttreten des Bundeskinderschutzgesetzes im Jahr 2012 und das Kinder- und Jugendstärkungsgesetz im Jahr 2021 [28]. Dennoch besteht nach wie vor viel Potenzial, um Wissen und Kompetenzen bei Mitarbeitenden in Krankenhäusern so zu verbessern, dass in Verdachtsfällen adäquat gehandelt werden kann.

Darüber hinaus hat sich in der vorliegenden Studie gezeigt, dass zwar in einigen Krankenhäusern schon vollständige ISK etabliert wurden, in den meisten Einrichtungen bisher aber lediglich einzelne Elemente umgesetzt wurden und ein Gesamtkonzept fehlt. Dies deckt sich mit den Ergebnissen eines Monitorings des Deutschen Jugendinstituts (DJI) aus den Jahren 2016/2017, welches zeigte, dass lediglich 20,1 % der betrachteten Kliniken ein umfassendes ISK entwickelt haben, eine Einzelmaßnahme hatten mindestens 56,7 % umgesetzt [13]. Die Literatur verdeutlicht aber auch, dass Personalausstattung und Vergütung von Kliniken im Hinblick auf die ISK-Entwicklung verbessert werden müssen [16, 29, 30]. Bezugnehmend auf die Regelungen zu ISK des G‑BA aus dem Jahr 2020 sollte die Anzahl der Krankenhäuser, die vollständige Konzepte etabliert haben, in den kommenden Jahren steigen. Dies lässt sich unterstreichen, wenn man sich ansieht, welche Elemente bereits vermehrt in medizinischen Einrichtungen realisiert wurden, da vor allem diejenigen ISK-Elemente in vielen Einrichtungen umgesetzt wurden, die verpflichtend vorgegeben sind, wie zum Beispiel das Einholen eines EFZ. Des Weiteren wurde in den Ergebnissen dieser Arbeit deutlich, dass Beschwerdeverfahren, Fortbildungen und ein Interventionsplan zu den am häufigsten realisierten ISK-Elementen zählen. Das Monitoring des DJI zu Schutzkonzepten im Gesundheitsbereich konnte die Risiko- und Potenzialanalyse, Verhaltenskodizes, Beschwerdeverfahren, Fehlermanagement, Aufklärung über Kinderrechte, die Partizipation von Kindern, Jugendlichen und Eltern, Personalauswahl und Führungszeugnis, Fortbildungen, Kooperation und Vernetzung sowie den Umgang mit digitalen Medien als Schutzkonzeptelemente in Krankenhäusern eruieren. Die Untersuchung erhob allerdings keine Häufigkeiten der Umsetzung der einzelnen Elemente [13]. Diese Unregelmäßigkeiten in der Umsetzung von ISK-Elementen in Krankenhäusern in Deutschland verdeutlichen, dass neben der Notwendigkeit einer Verpflichtung für eine medizinische Einrichtung zur Entwicklung eines ISK Regelungen zur Umsetzung und Finanzierung eines so komplexen und ressourcenintensiven Vorhabens in Fachbereichen mit Fachkräfte- und Personalmangel sowie hoher Arbeitsbelastung noch ausstehen. Hier ist es unbedingt wünschenswert, dass die entsprechenden Einrichtungen Unterstützung finanzieller und personeller Art erhalten und somit künftig neben der Quantität auch die Qualität von ISK in medizinischen Einrichtungen verbessert wird.

Der Umsetzungsgrad in der Praxis und die Akzeptanz der einzelnen ISK-Elemente wurden in der vorliegenden Untersuchung von den Befragten überwiegend mittelmäßig bis hoch eingeschätzt, ließen aber noch Potenzial erkennen, die ISK-Entwicklung in Krankenhäusern zu verbessern. Deshalb kann es sinnvoll sein, dass Krankenhäuser ihre Mitarbeitenden bei der Entwicklung eines ISK und dessen Elemente umfassend einbinden und versuchen, alle Bereiche miteinzubeziehen. Die Akzeptanz des ISK kann so verbessert werden. Zudem sollte eingeplant werden, dass die Entwicklung eines ISK ein langer und intensiver Prozess ist, der Personal und Zeit beansprucht. Es empfiehlt sich daher, eine ISK-Gruppe ins Leben zu rufen, die sich diesen Herausforderungen stellt und dafür entsprechende Ressourcen zur Verfügung gestellt bekommt. Bei der aktuell schwierigen personellen Situation im medizinischen Bereich können diese Ressourcen allerdings realistischerweise nur durch Unterstützung des Trägers oder aus öffentlicher Hand zur Verfügung gestellt werden.

### Stärken und Limitationen

Die vorliegende Studie weist Limitationen auf, vor allem angesichts der Zusammensetzung der nichtrepräsentativen Stichprobe aus Krankenhausmitarbeitenden, die an einem von 2 Online-Kursen teilnahmen. Zum einen kann nicht ausgeschlossen werden, dass Teilnehmende desselben Krankenhauses einen der Online-Kurse belegten und somit die einrichtungsspezifischen Fragen im Hinblick auf eine Einrichtung mehrfach beantwortet wurden. Die Ergebnisse können somit nicht im Hinblick auf die entsprechende Einrichtung, sondern nur auf die befragten Mitarbeitenden der Einrichtungen interpretiert werden. Zum anderen kann davon ausgegangen werden, dass Personen, die sich verstärkt für das Thema Kindesmisshandlungen, Kinderschutz und ISK interessieren, vermehrt an den Kursen teilnahmen. Diese Personen besitzen eventuell schon ein größeres Wissen und höhere Kompetenzen bezüglich des Umgangs mit Fällen von Kindesmisshandlung, sind eher mit entsprechenden Schutzmaßnahmen vertraut und nehmen Fälle von Gewalt eher wahr. Hierdurch kann es zu einem Selektionsbias kommen. Dieser kann sich noch dadurch verstärken, dass Teilnehmende aus Kliniken für Kinder- und Jugendpsychiatrie, -psychotherapie oder -psychosomatik mehr als die Hälfte der Befragten darstellen und diese vermutlich in ihrem Arbeitsalltag häufiger mit dem Thema Kindesmisshandlungen in Kontakt kommen. Zudem handelt es sich bei den Daten um retrospektive Selbsteinschätzungen der Befragten, diese könnten im Hinblick auf das Auftreten von Fällen in der eigenen Einrichtung einem Recall-Bias unterliegen. Des Weiteren ist nicht bekannt, wie lange die Befragten bereits in der Einrichtung arbeiten, was einen Einfluss auf die Anzahl der wahrgenommenen Fälle von Kindesmisshandlung haben kann. Die genannten Punkte müssen bei der Interpretation der Daten beachtet werden.

Die Stärke dieser Studie besteht in der Befragung von über 1000 Mitarbeitenden in Krankenhäusern deutschlandweit zum Thema Kinderschutz und ISK in der eigenen Einrichtung. Der Grad der Umsetzung von ISK-Elementen und deren Akzeptanz wurden außerdem in Deutschland bisher erst vereinzelt beleuchtet. Dies kann ein erstes Bild davon geben, wie Krankenhäuser in Deutschland im Hinblick auf Kinderschutz aufgestellt sind.

## Fazit

Es zeigte sich, dass sowohl Wissen und Kompetenzen im Umgang mit Kindesmisshandlung unter Mitarbeitenden in Krankenhäusern in Deutschland als auch ISK in Krankenhäusern zwar oftmals vorhanden sind, allerdings nicht besonders stark ausgeprägt bzw. weit gediehen sind. Die meisten Krankenhausmitarbeitenden schätzten ihr Wissen und ihre Kompetenzen mittelhoch ein, dies ist vor allem im Hinblick darauf alarmierend, dass die Befragten bereits ein erhöhtes Interesse für die Thematik zeigten. Lediglich ein Prozent der Befragten gab an, dass in der eigenen Einrichtung bereits alle Elemente eines ISK entwickelt wurden, 6,4 % gaben an, dass noch kein einziges Element entwickelt wurde. Die Regelungen des G‑BA zum ISK sind also bisher noch nicht in der Praxis angekommen. Allerdings werden weitere Studien benötigt, die systematisch erfassen, inwieweit Elemente eines ISK in Krankenhäusern in Deutschland umgesetzt und realisiert werden.
